# Convergent Early Dysregulation of Sarcopenia-Associated Genes in Skeletal Muscle During Aging and SOD1G93A ALS Models

**DOI:** 10.3390/ijms27219529

**Published:** 2026-10-25

**Authors:** Paula Aparicio, Gabriella Dobrowolny, Martina Lupoli, Laura Moreno-Martínez, Tresa López-Royo, Antonio Musarò, Raquel Manzano, Rosario Osta

**Affiliations:** 1 LAGENBIO, Faculty of Veterinary, University of Zaragoza, Miguel Servet 177, 50013 Zaragoza, Spain; apariciop@unizar.es (P.A.); lauramm@unizar.es (L.M.-M.); tlopez@unizar.es (T.L.-R.); rmanzano@unizar.es (R.M.); 2 Centre for Biomedical Research in Neurodegenerative Diseases (CIBERNED), Instituto de Salud Carlos III, 28029 Madrid, Spain; 3 AgroFood Institute of Aragon (IA2), 50013 Zaragoza, Spain; 4 Institute of Health Research of Aragon (IIS), 50009 Zaragoza, Spain; 5 Laboratory Affiliated to Istituto Pasteur Italia—Fondazione Cenci Bolognetti, Sapienza University of Rome, 00161 Rome, Italy; gabriella.dobrowolny@uniroma1.it (G.D.); martina.lupoli@uniroma1.it (M.L.); antonio.musaro@uniroma1.it (A.M.); 6 Scuola Superiore di Studi Avanzati Sapienza (SSAS), Sapienza University of Rome, 00161 Rome, Italy

**Keywords:** ALS, sarcopenia, aging, skeletal muscle, atrophy

## Abstract

Skeletal muscle undergoes structural and functional decline due to aging and neuromuscular disorders such as amyotrophic lateral sclerosis (ALS), both of which impact protein expression and contribute to muscle atrophy. This study investigates how aging and the SOD1^G93A^ ALS-related mutation affect the expression of eight genes previously linked to sarcopenia (PF4, OIT3, MMRN1, MASP1, C1R, SVEP1, VCAN and SPTB) but not yet characterized in muscle tissue. To this end, we analyzed their expression (1) during muscle aging in WT mice (5 and 24 months), (2) in SOD1^G93A^-transfected myotube cells in vitro, (3) in a muscle-specific SOD1^G93A^ mouse model (5 and 24 months), and (4) in a systemic SOD1^G93A^ transgenic mouse at different disease stages (60 and 120 days) by quantitative PCR. All eight target genes were expressed in skeletal muscle and most of them showed increased transcription with aging. In ALS models, gene expression was significantly overexpressed at early stages, particularly in muscle-specific (MLC/SOD1^G93A^) and systemic (SOD1^G93A^) models, mimicking aging-related profiles. These alterations attenuated with disease progression, suggesting an early transient muscle response to ALS-linked SOD1 mutations. Hence, this study demonstrates that sarcopenia-associated proteins are actively expressed and modulated in skeletal muscle during aging and ALS and suggests convergent response mechanisms.

## 1. Introduction

Skeletal muscle is a dynamic tissue that plays a crucial role in physical functions, encompassing locomotion, gesture support, and everyday life processes. Furthermore, these muscles serve as the primary tissue for energy metabolism, engaging in the absorption, application, and storage of energy substrates like glucose, fats, and amino acids. Its structural and functional integrity is precisely regulated by a complex network of biomolecules, such as proteins, whose expression profiles change physiologically throughout life but also in response to pathological conditions. The decline in muscle mass, strength and performance is referred to as muscle atrophy and this deterioration adversely impacts the daily living of patients, diminishing the capacity to perform daily tasks and prolonging the healing process after an illness. Among the factors that contribute to muscle degeneration, we can differentiate congenital or genetic causes, called primary skeletal muscle atrophy, and acquired causes secondary to either physiological or pathological conditions. Among secondary skeletal muscle atrophy, aging and neuromuscular disorders are particularly impactful as secondary physiological and pathological causes of muscle deterioration, respectively.

Aging involves a series of physiological changes in different systems of the organism, including the musculoskeletal system [1]. When the originally progressive decline in muscle mass and function occurs abruptly and results in adverse consequences for the patient it is considered a disease called sarcopenia [2]. This disorder is accompanied by cellular and molecular processes in muscle fibers, namely, the transformation of the fiber class from type II (fast twitch and anaerobic metabolism) to type I (slow twitch and aerobic metabolism) [3], an accumulation of cell damage due to decreased autophagy mechanism [4], the decreased function and energy produced in mitochondria [5], the infiltration of adipocytes in inter- and intra-muscular tissue [6], or the alteration or mismatch of the coupled excitation–contraction process [7]. Dysregulation of satellite cells (undifferentiated stem cells in muscle tissue), which contribute to muscle maintenance and regeneration, may also play a role in this process, while this dysregulation does not necessarily involve a reduction in satellite cell number: impaired activation, proliferation, differentiation, or self-renewal may also compromise muscle regenerative capacity [8].

Age-related skeletal muscle decline occurs in the context of broader biological processes described as the hallmarks of aging, which include chronic low-grade inflammation (inflammaging) [9,10], altered intercellular communication, and progressive extracellular matrix (ECM) remodeling [11]. These mechanisms contribute to impaired regenerative capacity, increased fibrosis, and functional deterioration of skeletal muscle during aging [11,12]. Interestingly, similar processes have also been described in neuromuscular disorders such as amyotrophic lateral sclerosis (ALS) [13], suggesting that partially overlapping molecular programs may operate in both physiological aging and neurodegenerative muscle degeneration. ALS is a lethal neurodegenerative condition characterized by the gradual degeneration of both upper and lower motor neurons, resulting in muscle weakness, atrophy, and ultimately death. While it has traditionally been perceived as a motoneuron disease, growing evidence suggests that skeletal muscle is not merely a passive target but an active contributor to disease development [14,15]. However, the specific gene-level changes occurring in muscle tissue during aging and under ALS-like conditions remain poorly characterized.

A previous study revealed eight proteins whose concentration was significantly different in plasmatic extracellular vesicles obtained from sarcopenic and robust individuals [16]. Four of these eight proteins were lower in sarcopenic patients: Platelet factor 4, also known as CXCL4 (PF4), Oncoprotein-induced transcript 3 protein (OIT3), Multimerin-1 (MMRN1) and Mannan-binding lectin serine protease 1 (MASP1); and four showed higher concentration: Complement C1r subcomponent (C1R), Sushi von Willebrand factor type A, EGF and pentraxin domain-containing protein 1 (SVEP1), Versican core protein (VCAN), and Spectrin beta chain, erythrocytic (SPTB). Limited studies investigate the expression of these proteins in muscle during aging and during the course of skeletal muscle altering diseases such as ALS. In fact, some of these proteins, like OIT3, have never been related to muscle physiology or pathology and its expression has never been identified in this tissue.

**Figure 1:**
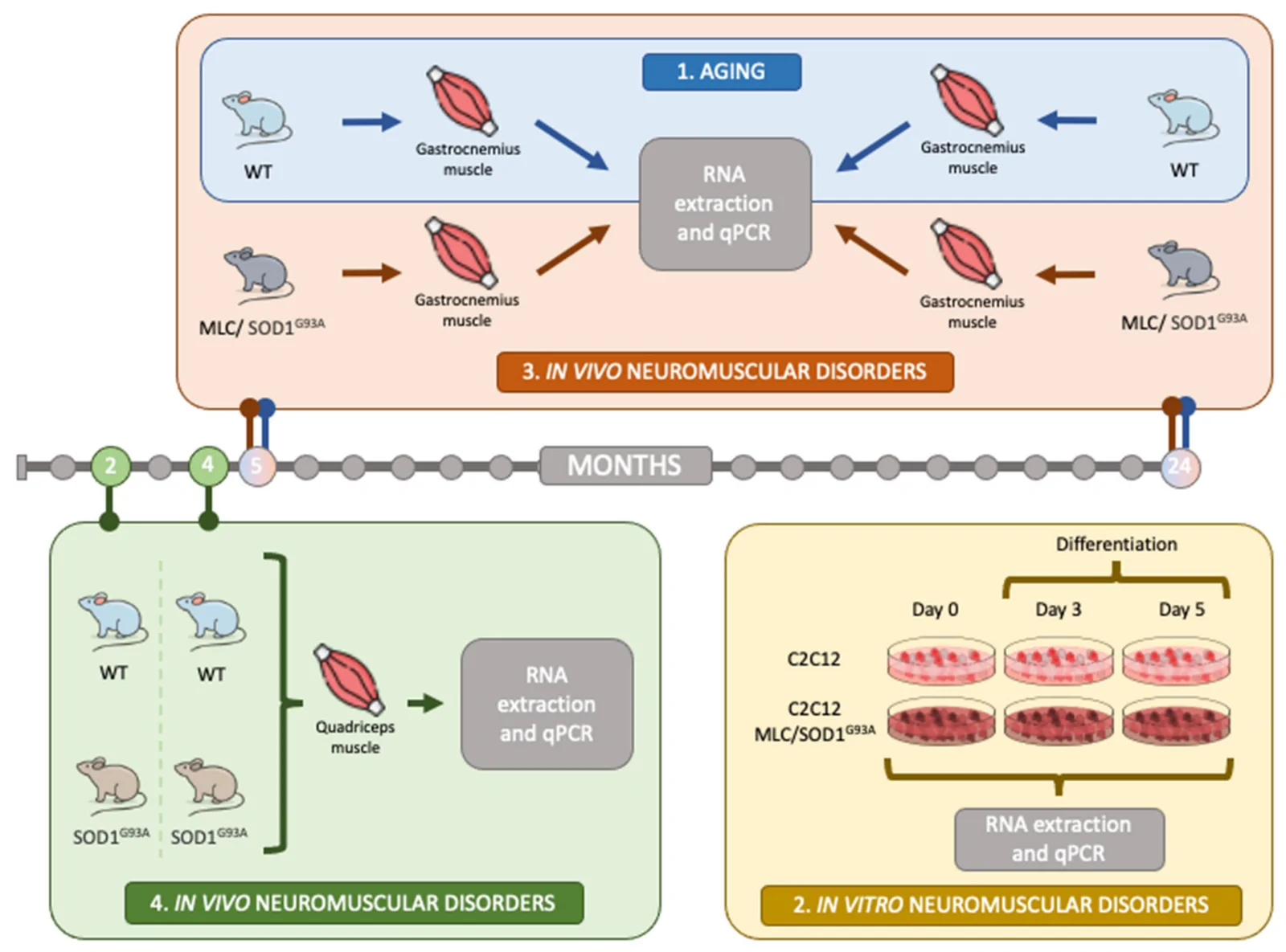
**Graphical scheme of the assays.** Experimental design for qPCR quantification of target genes during aging, using wild-type (WT) mice at the age of 5 and 24 months (Assay 1), and during muscle dysfunction, using several experimental models: in the cellular culture of muscle C2C12 MLC/SOD1^G93A^ during 5 days of myogenic differentiation (Assay 2); in the murine model MLC/SOD1^G93A^ that expresses a mutant SOD1^G93A^ gene selectively in skeletal muscle using WT and transgenic mice at the age of 5 and 24 months (Assay 3) and in the SOD1^G93A^ transgenic mice that overexpress a mutant SOD1 gene ubiquitously using WT mice at the age of 2 and 4 months (Assay 4). Target genes: PF4, OIT3, MMRN1, MASP1, C1R, SVEP1, VCAN, SPTB; qPCR: quantitative PCR.

Therefore, this study aimed to determine whether sarcopenia-associated genes identified in circulating extracellular vesicles, hereon defined as our target genes, are also transcriptionally regulated in skeletal muscle during aging and in ALS-related models, as shown in Figure 1. We hypothesized that aging and ALS-associated muscle degeneration may partially converge on an early shared molecular program involving inflammatory signaling, extracellular matrix remodeling, and tissue stress responses.

## 2. Results

### 2.1. All Target Genes Were Expressed in Skeletal Muscle

We first studied whether all of the target genes were expressed in skeletal muscle. The eight target genes showed expression in WT mouse hindlimb muscles, given that their CTs, in most cases, were below 27, as can be observed in Table A1. Moreover, a single peak on the melt curve was checked in all of the cases and the annealing temperatures of the primers were optimized with an efficiency that was always above 90%. Therefore, we proceeded with the subsequent experiments.

### 2.2. Expression of Target Genes Increased in Aged Gastrocnemius Muscles

In order to study whether the expression of target genes was modified during aging in skeletal muscle, we compared the relative expression of each one in the gastrocnemius muscles of WT mice at the age of 5 and 24 months.

**Figure 2:**
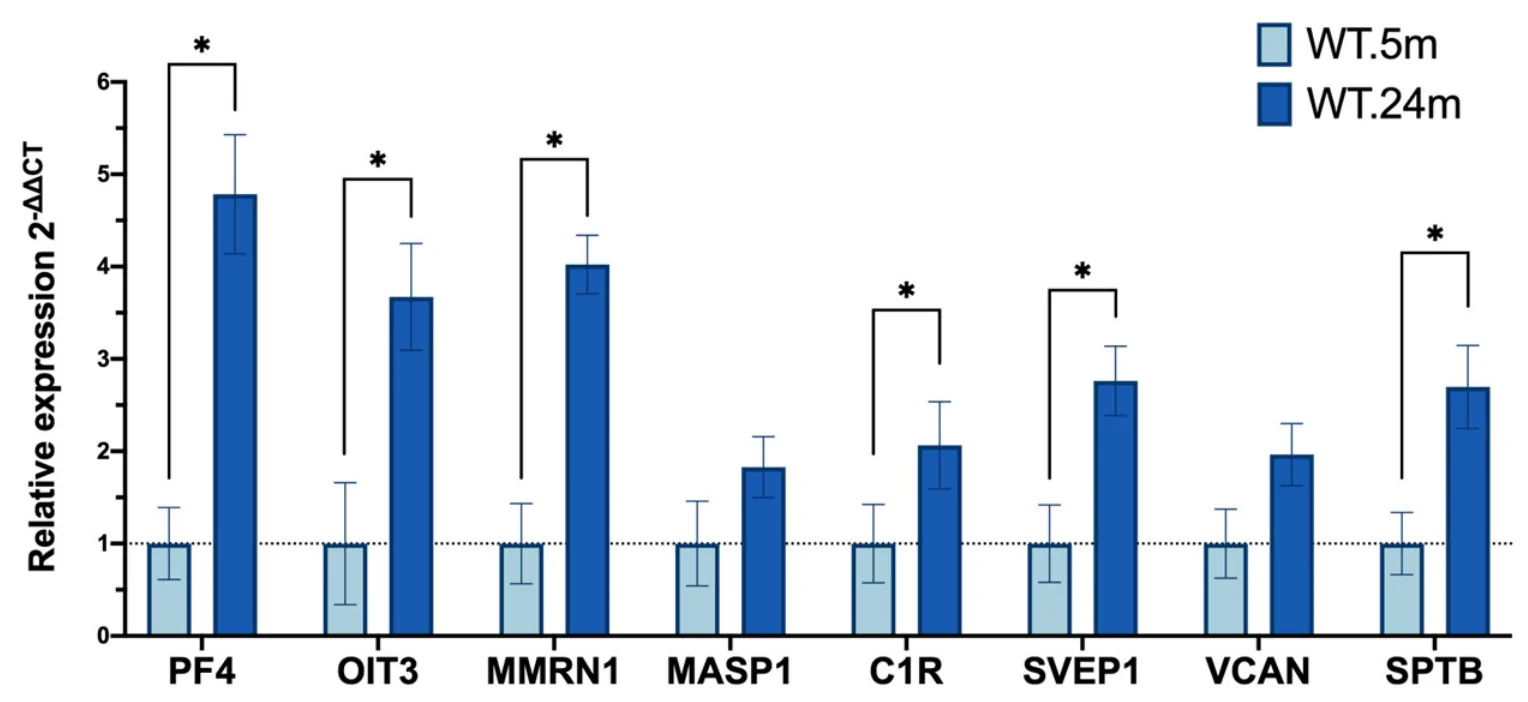
**Gene expression of PF4, OIT3, MMRN1, MASP1, C1R, SVEP1, VCAN and SPTB in the gastrocnemius muscles of three 5-month-old wild-type mice (WT.5m) and three 24-month-old wild-type mice (WT.24m) from Assay 1.** Expression levels were evaluated by quantitative PCR and parametric Student’s *t*-tests were used to compare the groups. *p* ≤ 0.05 (*).

Transcriptional levels of all genes show an increase with age (Figure 2 and Table A1 and Table A2); this rise was statistically significant in all of them except *MASP1* and *VCAN*. The highest increases were observed for *PF4* (*p* = 0.013), *MMRN1* (*p* = 0.022) and *OIT3* (*p* = 0.013) with Log2 fold changes of 2.258, 2.008 and 1.877 respectively, followed by *SVEP1* (*p* = 0.030, Log2FC = 1.466), *SPTB* (*p* = 0.044, Log2FC = 1.431) and *C1R* (*p* = 0.027, Log2FC = 1.046). Finally, the levels of the *MASP1* and *VCAN* transcripts also show a trend toward an increase in aged mice, although this difference does not reach statistical significance.

### 2.3. Target Genes Are Affected by Myogenic Differentiation and Genotype in C2C12 MLC/SOD1^G93A^ Culture

As a second objective, we proposed to study how mutation in the superoxide dismutase 1 (SOD1) gene, one of the most extensively studied mutations in the context of the genetic form of the neuromuscular disorder ALS, affects the expression of these transcripts in skeletal muscle in different in vitro and in vivo models. In order to assess this objective, we initially studied the expression of the target genes using an in vitro model transfected with SOD1 gene (SOD1^G93A^) plasmids, comparing these expressions in the transfected cells (C2C12 MLC/SOD1^G93A^ cells (CSOD) and in control culture (C2C12) during five days of myogenic differentiation.

**Figure A1:**
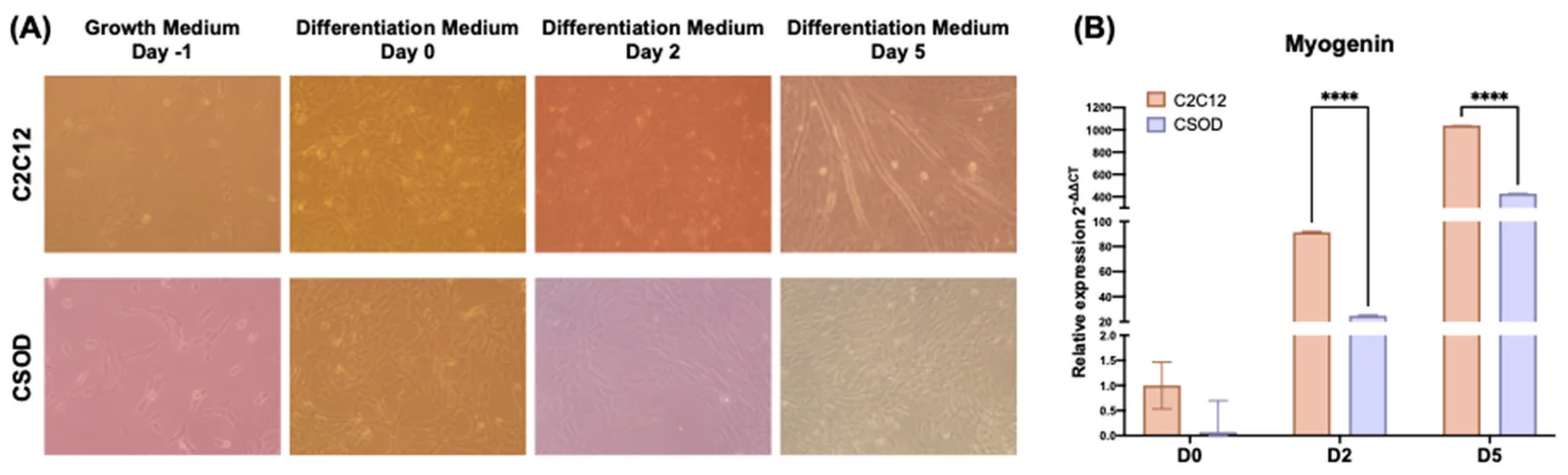
**Study of the phenotype and transcriptional myogenin levels in C2C12 cultures transfected with SOD1^G93A^ plasmids and controls during myogenic differentiation.** Mouse C2C12 MLC/SOD1^G93A^ (CSOD) and control (C2C12) cells were plated at the same density and maintained under the same conditions. At day 0, cells were shifted to differentiation medium in order to induce myogenic differentiation. (**A**) Cells were photographed and harvested at day 0 (D0), day 2 (D2; 48 h after shift in DM), and day 5 (D5; 120 h after shift in DM). Then, RNA was extracted and used in subsequent PCR against different primers, in the graph shown, against myogenin (**B**). *p* ≤ 0.0001 (****).

Firstly, we confirmed that SOD1 mutant expression impaired myogenic differentiation in CSOD cells, showing no multinucleated fibers and markedly reduced myogenin levels when compared with controls, consistent with Martini et al. (2015) [17], as can be observed in Figure A1.

**Figure 3:**
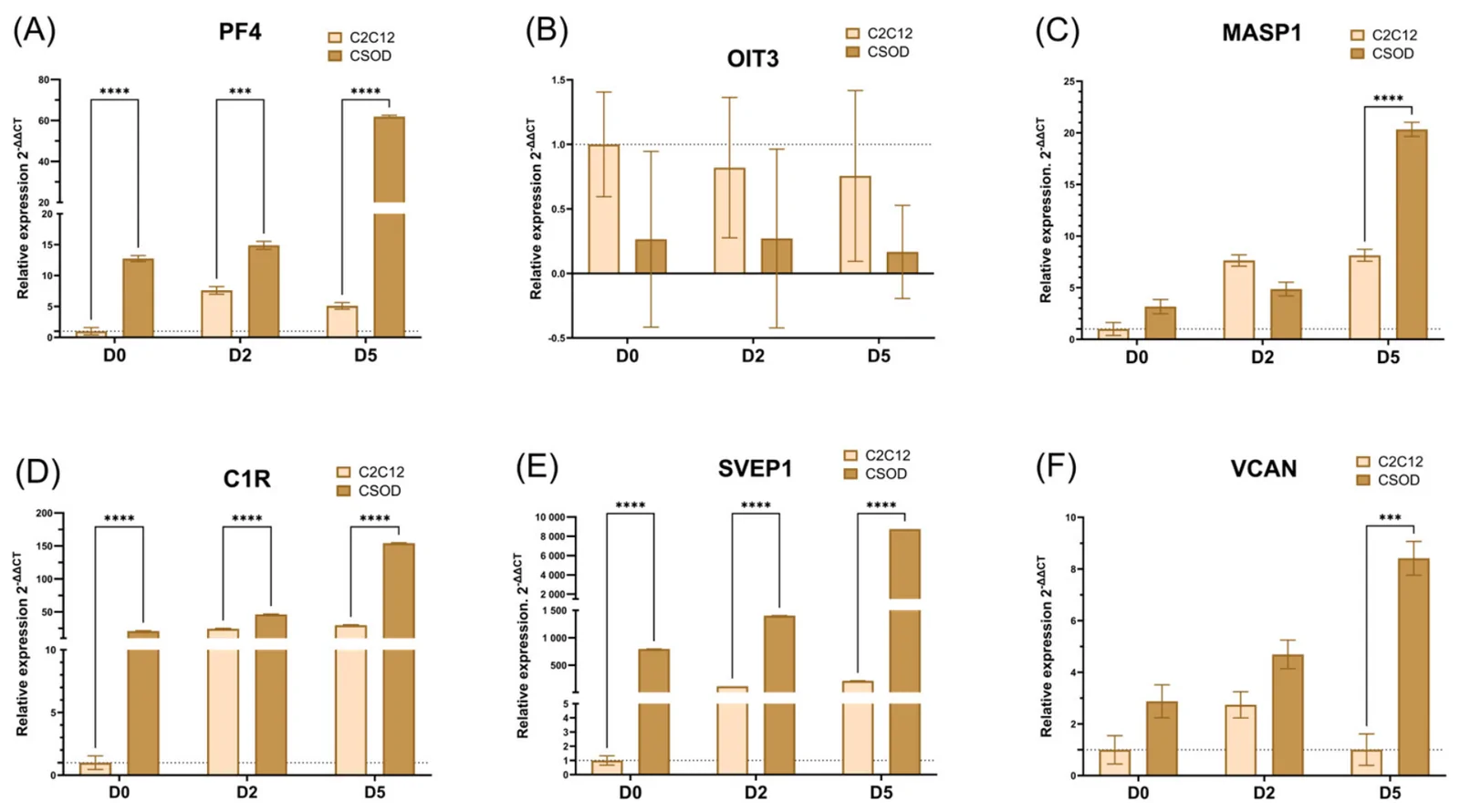
**Bar graphs of transcriptional levels of PF4 (A), OIT3 (B), MASP1 (C), C1R (D), SVEP1 (E) and VCAN (F) in C2C12 cultures transfected with SOD1G93A plasmids and controls during myogenic differentiation in Assay 2 (*n* = 3).** Mouse C2C12 MLC/SOD1^G93A^ (CSOD) and control (C2C12) cells were plated at the same density and maintained under the same conditions. At day 0, cells were shifted to differentiation medium in order to induce myogenic differentiation (*n* = 3 per condition and stage). Cells were harvested at day 0 (D0), day 2 (D2), and day 5 (D5) and RNA was extracted and used in subsequent qPCR against different primers. *p* ≤ 0.001 (***) and *p* ≤ 0.0001 (****).

When studying the expression levels of *PF4*, *OIT3*, *MMRN1*, *MASP1*, *C1R*, *SVEP1*, *VCAN* and *SPTB* genes, we first noticed that *MMNR1* and *SPTB* genes were not amplified in any of the cell cultures or degrees of differentiation. For the six genes for which we did find expression (*PF4*, *OIT3*, *MASP1*, *C1R*, *SVEP1*, *VCAN*), the transcriptional levels were higher in the transfected culture for all genes and stages of differentiation except for *MASP1* on the second day of differentiation and of *OIT3*, where the levels were higher in the control cultures throughout differentiation, although this difference was not statistically significant in either of these two exceptions (Figure 3, Table A3). With the exception of *OIT3*, both genotype and differentiation factors were significant in the two-way ANOVA analysis and, in all cases, there was an interaction between both. Gene deregulation remained most significant at day 5 of differentiation, except in OIT3.

### 2.4. Young MLC/SOD1^G93A^ Mice Show Increased Expression of Target Genes

Subsequently, we intended to study the expression of our genes in a model of ALS with mainly muscular involvement, so we used the MLC/SOD1^G93A^ murine model that expresses a mutant SOD1^G93A^ gene selectively in skeletal muscle.

**Figure 4:**
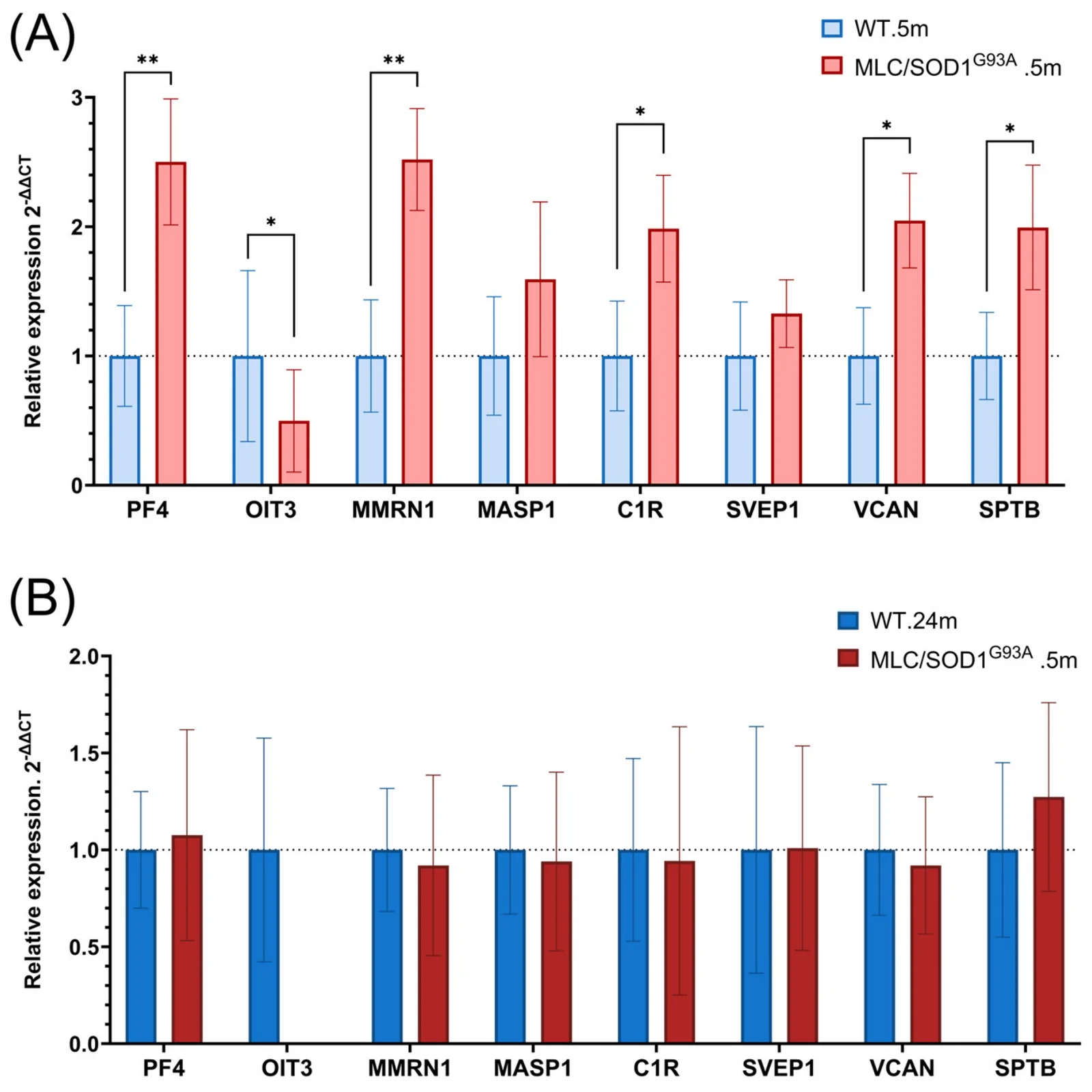
**Bar plot of the expression levels of the target genes in the skeletal muscle of 5-month-old and 24-month-old WT and mSOD mice from Assay 3.** At the age of 5 (**A**) and 24 months (**B**), 3 male mice of each genotype and age were sacrificed and RNA from gastrocnemius muscles was extracted for subsequent qPCR analysis. Parametric Student’s *t*-tests were used to compare the groups. WT.5m: 5-month-old wild-type mice; MLC/SOD1^G93A^.5m: 5-month-old MLC/SOD1^G93A^ mice; WT.24m: 24-month-old wild-type mice; MLC/SOD1^G93A^.24m: 24-month-old MLC/SOD1^G93A^ mice. *p* ≤ 0.05 (*), *p* ≤ 0.01 (**).

When studying gene expression in 5-month-old mice, we observed that most genes are increased in MLC/SOD1^G93A^ mice compared with WT mice. Specifically, we can find a statistically significant upregulation in PF4 (*p* = 0.008), MMRN1 (*p* = 0.007), C1R (*p* = 0.041), VCAN (*p* = 0.033) and SPTB (*p* = 0.039). The only downregulated gene in MLC/SOD1^G93A^ mice at 5 months of age compared with WT is OIT3, with a significance of *p* = 0.04001 (Figure 4A).

On the other hand, when comparing MLC/SOD1^G93A^ and WT mice at the age of 24 months, we found no significant differences in the expression of these genes (Figure 4B), while it is necessary to mention that we could not find any amplification of OIT3 expression levels at 24 months old in MLC/SOD1^G93A^ mice.

The raw data for the cycles of all analyses are shown in Table A1, while the specific data and statistical results of this comparison are detailed in Table A4.

### 2.5. Variation in the Expression of the Target Genes Occurs at the Pre-Symptomatic Stage in Ubiquitous SOD1^G93A^ Mice

Lastly, we sought to investigate possible dysregulation in the expression of target genes when introducing the motoneuron impairment in the skeletal muscle using the SOD1^G93A^ transgenic mice, an ALS model that overexpresses a mutated SOD1 gene ubiquitously. In particular, we compared the results of WT and SOD1^G93A^ transgenic mice at 60 and 120 days, corresponding to the pre- and late symptomatic stages respectively.

**Figure 5:**
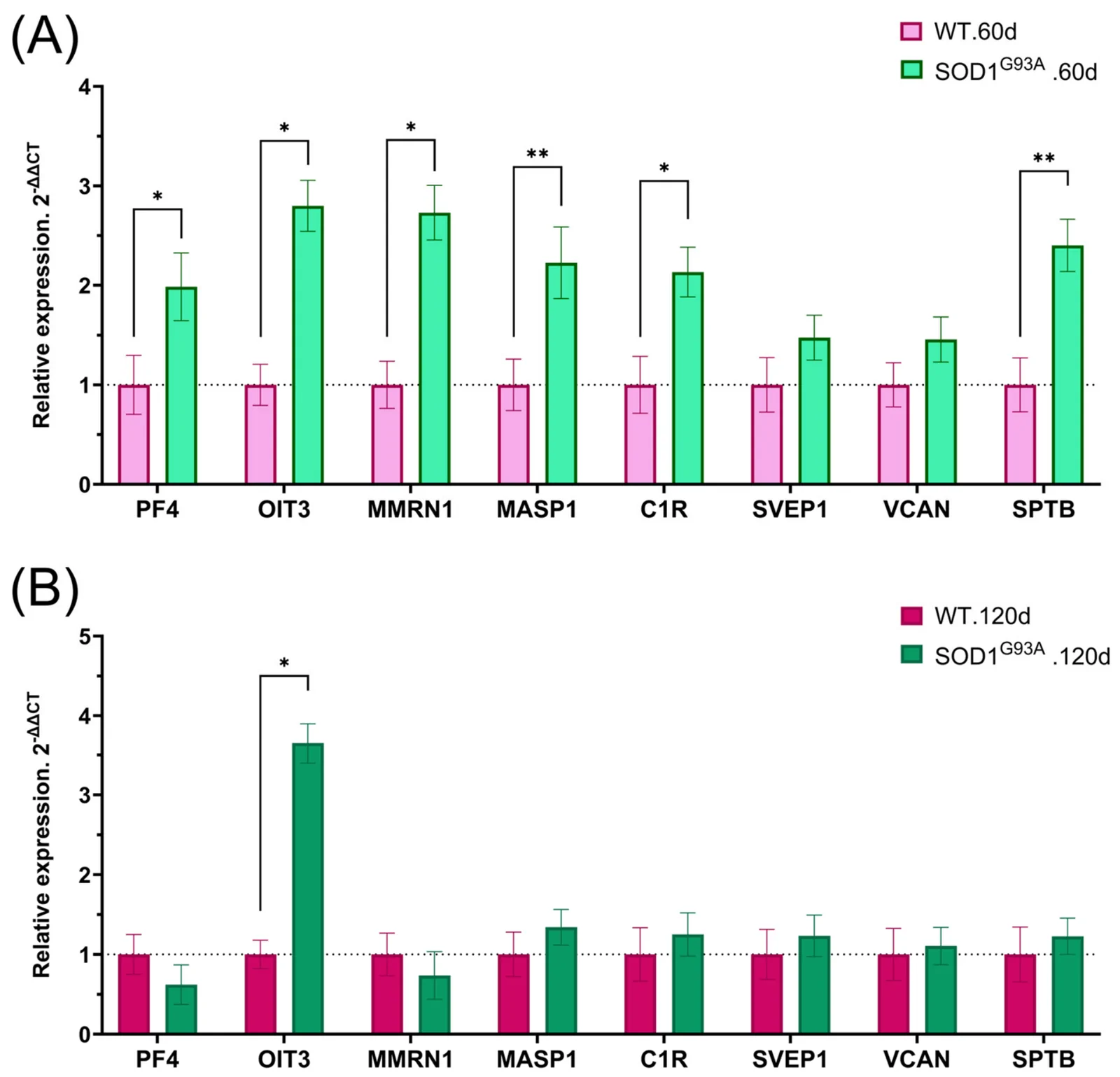
**Bar plot of the expression levels of the target genes in the skeletal muscle of WT and SOD1^G93A^ mice from Assay 4 at 60 (A) and 120 days (B), as they correspond to the pre-symptomatic and late symptomatic stages, respectively.** Parametric Student’s *t*-tests were used to compare the groups (*n* = 6 male mice per genotype and age). WT.60d: 60-day-old wild-type mice; SOD.60d: 60-day-old SOD1^G93A^ mice; WT.120d: 120-day-old wild-type mice; SOD.120d: 120-day-old SOD1^G93A^ mice. *p* ≤ 0.05 (*), *p* ≤ 0.01 (**).

Starting with the comparison between groups of mice in the pre-symptomatic state, i.e., at 60 days of age, we observed an increase in the expression of all genes in mice carrying the mutation compared with the WT controls (Figure 5A). This increase is statistically significant in PF4 (*p* = 0.038), OIT3 (*p* = 0.022), MMRN1 (*p* = 0.025), MASP1 (*p* = 0.006), C1R (*p* = 0.0258) and SPTB (*p* = 0.005). At the terminal stage of the SOD1G93A mice, corresponding with the age of 120 days, no significant difference in gene expression is observed, except for OIT3, which continues to significantly increase in the transgenic mouse vs. the WT at 120 days (*p* = 0.014) (Figure 5B). The complete cycle data for all analyses are provided in Table A1, and the corresponding detailed results and statistical comparisons are presented in Table A5.

**Figure 6:**
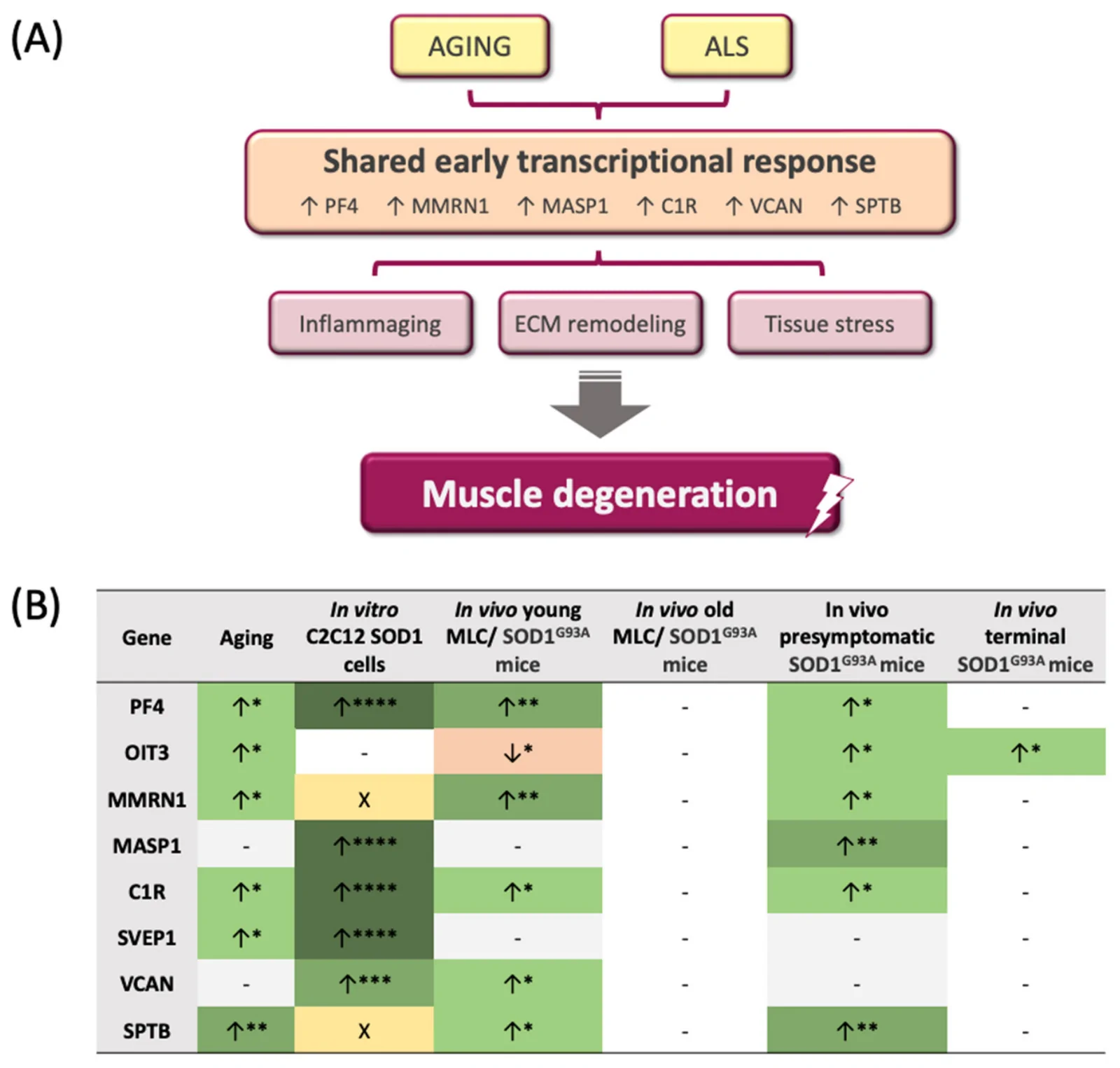
**Shared transcriptional signature of sarcopenia-related genes across aging and ALS models.** (**A**) Conceptual model illustrating the proposed early shared molecular program activated in skeletal muscle during aging and ALS. Aging-related processes and SOD1-associated neurodegeneration converge in a transcriptional response involving inflammation, extracellular matrix remodeling, and tissue stress pathways. (**B**) Heatmap summarizing the regulation of sarcopenia-related genes across experimental models used in this study. X, no amplification; -, no statistical variation; *p* ≤ 0.05 (*), *p* ≤ 0.01 (**), *p* ≤ 0.001 (***) and *p* ≤ 0.0001 (****).

Figure 6 presents an integrated transcriptional signature summarizing the regulation of these genes in aging and ALS models, allowing visualization of their coordinated behavior across experimental systems.

## 3. Discussion

Aging-related muscle loss, known as sarcopenia, and ALS are two distinct processes that culminate in skeletal muscle degeneration. Despite differences in primary cause, prevalence or speed of progression, sarcopenia and ALS share some overlapping features regarding the skeletal muscle such as denervation, neuromuscular junction (NMJ) destabilization, fiber-type specific vulnerability or mitochondrial dysfunction. A previous study conducted in our laboratory identified eight proteins dysregulated in plasma extracellular vesicles of sarcopenic individuals (PF4, OIT3, MMRN1, MASP1, C1R, SVEP1, VCAN, and SPTB) [16]. Given that sarcopenia is a disease that primarily affects skeletal muscle, we hypothesize that the dysregulation of these eight proteins in plasma extracellular vesicles must be related to failures in muscle homeostasis mechanisms. For this reason, we compared the expression patterns of these target genes in skeletal muscle across different experimental models of aging in order to better understand how aging and ALS-related mutations may converge in modulating muscle homeostasis.

We first found that all sarcopenia-related genes are actively transcribed in mouse skeletal muscle tissue, which is, to the best of our knowledge, the first report for some of these genes such as OIT3 and MMRN1 in this tissue. Subsequently, we observed a general upregulation of these transcripts in 24-month old mouse muscle, consistent with previous transcriptomic and proteomic analyses showing an increase in some of these genes with aging in plasma and mesenchymal stem cells (MSCs) [18,19,20,21]. Hao et al. in 2024 [19] identified VCAN among the top six overlapping hub genes that were significantly altered in MSCs from aged donors compared with young donors. Quantitative PCR validation confirmed that VCAN expression was significantly upregulated in in vitro-induced senescent MSCs compared with control cells, supporting its utility as a molecular marker of MSC aging [19]. In 2019, a study measured 2925 plasma proteins in 4263 individuals aged 18 to 95 years, using the SomaScan platform and discovered an age-related upregulation of proteins involved in structural or extracellular matrix (ECM) pathways, such as SVEP1. SVEP1 is an ECM protein involved in cell adhesion, integrin α9β1 signaling and vascular homeostasis and its rise in late life likely reflects general and progressive tissue remodeling, chronic vascular inflammation, and ECM stiffening, hallmarks of advanced aging [18]. Aged muscle is characterized by chronic low-grade inflammation or *inflammaging* and a remodeling of ECM components that impairs fiber regeneration and contractility [9,22], which is consistent with SVEP1 upregulation. Furthermore, increased expression of PF4, a chemokine known to modulate angiogenesis and inflammatory responses [23], might reflect a compensatory response to endothelial dysfunction and immune infiltration observed during muscle aging. Similarly, C1R, a component of the classical complement pathway, and MASP1, part of the lectin pathway, participate in innate immune activation, which is increasingly recognized as a contributor to muscle degeneration and fibrosis [10,22].

Traditionally, ALS was viewed as a motoneuron-centric disease, but increasing evidence indicates that skeletal muscle contributes to the disease’s onset and progression [14,15,24]. Therefore, we wanted to study the effect of the SOD1 mutation, one of the causes of familial ALS, on the expression of target genes in skeletal muscle in different animal models. For the in vitro experiments we used C2C12 MLC/SOD1^G93A^ cells, where postmitotic expression of the SOD1^G93A^ gene is known to alter muscle cell identity, affect cellular homeostasis, and inhibit muscle differentiation [17]. The results revealed that expression of the mutant SOD1 gene also caused transcriptional dysregulation of most target genes, with a tendency toward increased expression at later stages of differentiation (day 5), except for OIT3 and MASP1, which followed divergent trends. Based on these results, the genes studied could be related to the aforementioned mechanisms affected in the model, to which excessive oxidative stress and a triggering of myoblasts toward an adipogenic phenotype can be added [17].

Given that gene ontology biological processes across target genes are related to calcium ion binding, collagen-containing extracellular matrix (ECM) and humoral immune response [16], the transient activation of the studied sarcopenia-related genes at early stages may represent an initial compensatory response aimed at maintaining muscle integrity [25,26,27]. However, the transient upregulation of these genes may represent an early adaptive response aimed at preserving muscle integrity through inflammatory and extracellular matrix remodeling pathways. In the systemic SOD1^G93A^ mouse model, most target genes showed increased expression at the pre-symptomatic stage (60 days), whereas these differences were largely absent at the late symptomatic stage (120 days), with OIT3 being the only gene that remained significantly upregulated. These findings indicate that the transcriptional changes observed at the pre-symptomatic stage were attenuated as the disease progressed. However, the lack of statistically significant differences at 120 days does not necessarily demonstrate that the underlying biological processes were resolved, but rather that differences in the expression of most target genes were no longer detectable at this stage.

Muscle injury has been reported to lead to increased expression of target genes. In this sense, PF4 protein increased in the biceps femoralis of a 3-month-old Wistar rat at day 3 post muscle injury when compared with control rats that underwent a sham treatment, as measured by mass spectrometry [28]. This protein was related to leukocyte chemotaxis, wound healing, negative regulation of angiogenesis, blood vessel morphogenesis, and coagulation [28]. Furthermore, C1R expression may be associated with activation of the Wnt/β-catenin pathway after muscle injury, since transcripts for this gene were increased in the skeletal muscle of 2-month-old TOPGAL mice 2 days after cryoinjury [29]. TOPGAL mice are transgenic animals used to detect Wnt/β-catenin signaling activity in vivo through the expression of a β-galactosidase reporter with X-gal staining. On the other hand, the upregulation of ECM-associated genes such as SVEP1, C1R, and PF4 might also reflect ongoing tissue remodeling and cytoskeletal adaptation to cellular stress [30,31,32]. Excessive ECM reorganization and loss of satellite cell number/function reduce regenerative capacity and promote stiffness and functional decline [33]; these same features are found in sarcopenic muscle and documented in ALS-affected muscle [34]. In fact, studies have emphasized that systemic inflammation and skeletal muscle loss are both strong prognostic factors in amyotrophic lateral sclerosis (ALS), with reduced muscle mass and elevated inflammatory markers independently associated with poorer survival outcomes [35,36]. However, while these features are also characteristic of age-related sarcopenia [9,37], their coexistence in ALS could reflect convergent downstream consequences of chronic metabolic and catabolic stress, rather than identical upstream mechanisms. Thus, shared phenomena such as inflammation or extracellular matrix remodeling should be interpreted as parallel adaptive or degenerative responses to cellular stress. Further integrative studies are needed to determine whether overlapping molecular signatures in ALS and aging muscles represent true mechanistic convergence or context-dependent phenotypic similarities.

Interestingly, OIT3 showed downregulation in both the C2C12 MLC/SOD1^G93A^ cells and the skeletal muscle of young MLC/SOD1^G93A^ mice. Although OIT3 has not been previously studied in muscle, recent data suggest a role in lipid metabolism and hepatic extracellular matrix interactions [38,39]. It would be interesting to further investigate its possible involvement in the pathogenesis of aging and neuromotor diseases such as ALS.

An important limitation of this study is that the expression of the eight sarcopenia-associated genes was evaluated exclusively at the mRNA level using qPCR. In this regard, it is important to consider that gene expression in muscle and the abundance of proteins in circulating extracellular vesicles are distinct measures, obtained from different tissues and biological contexts. Therefore, they may not necessarily correlate or change in the same direction as those previously reported [16], as is the case of PF4, MMRN1, and MASP1. In fact, discrepancies between muscle gene expression and circulating protein abundance have also been reported in other muscle disorders. A recent study comparing serum protein levels with the expression of the corresponding mRNAs in skeletal muscle from individuals with Duchenne muscular dystrophy and healthy controls identified discordant changes between these two biological compartments [40]. For example, serum complement C3 levels were reduced despite increased C3 mRNA expression in skeletal muscle [40]. This may reflect differences in tissue-specific regulation, protein release, distribution, and clearance. Nevertheless, further studies directly assessing the corresponding proteins in muscle tissue and circulating extracellular vesicles are required to determine the mechanisms underlying these differences. Such validation would help clarify the functional relevance of these genes in skeletal muscle during aging and ALS progression.

In summary, this study shows that a group of sarcopenia-related genes, previously identified in plasma, are actively expressed and dynamically regulated in skeletal muscle in two conditions, aging and ALS. These genes display a biphasic expression pattern, characterized by early upregulation during aging and at pre-symptomatic ALS stages, followed by normalization or decline as degeneration progresses. The parallel upregulation of several target genes during physiological aging and at pre-symptomatic stages of ALS models suggests the activation of an early shared molecular response in skeletal muscle, such as inflammation, extracellular matrix remodeling, or angiogenesis. Rather than representing disease-specific pathways, these transcriptional changes may reflect a conserved stress-response program aimed at preserving tissue integrity in the face of metabolic, inflammatory, or neurogenic insults.

## 4. Materials and Methods

### 4.1. In Vitro C2C12 and C2C12 MLC/SOD1^G93A^ Cell Culture

C2C12 MLC/SOD1^G93A^ mice (generated and characterized by Martini et al. in 2015 [17], from now on CSOD) and C2C12 cells (RRID: CVCL_0188) were defrosted and maintained at a density of 15,000 cells/cm^2^ for 48 h at 37 °C and 5% CO^2^. Cells were grown in culture medium, consisting of Dulbecco’s modified Eagle’s medium (DMEM) with 4.5 g/L glucose and L-glutamine (5796, Sigma Aldrich, St. Louis, MO, USA), supplemented with 15% fetal bovine serum (Gibco, Grand Island, NY, USA), 100U/mL penicillin and 100 µg/mL streptomycin (P0781, Sigma Aldrich), and 2.5 µg/mL of puromycin (P9620, Sigma Aldrich) for C2C12 MLC/SOD1^G93A^ cells. After 48 h from seeding, on day 0, cells were shifted to differentiation medium (DM), consisting of DMEM (5796, Sigma Aldrich) with 2% horse serum (Gibco) in order to induce differentiation. Cells were harvested at day 0 (D0), day 2 (D2; 48 h after shift in DM), and day 5 (D5; 120 h after shift in DM) and RNA was extracted. Three technical qPCR measurements were performed for each one of the biological replicates.

### 4.2. In Vivo Animal Models

Several mouse lines were used to evaluate how atrophy and neuromuscular disorders, specifically ALS, affect the expression of these genes in muscle. Specific details on genotype, sex, and age of all of the mice employed in this research can be found in Table A6.

#### 4.2.1. MLC/SOD1^G93A^ Mice

MLC/SOD1^G93A^ mice, the transgenic mouse model characterized by Dobrowolny et al. in 2008 [14], selectively expresses a mutant SOD1 gene (SOD1^G93A^) in skeletal muscle and were thus used as a model for muscle atrophy and ALS. FVB mice (Jackson Laboratories; RRID: MGI:3609372) were used as wild-type (WT) mice. At the age of 5 and 24 months, 3 male mice of each genotype and age were sacrificed, and gastrocnemius muscles were harvested, flash-frozen in liquid nitrogen and stored at −80 °C. The animals were housed in a temperature-controlled (22 °C) room with a 12 h light–dark cycle. All mice were maintained in compliance with the institutional guidelines of the DIEM Animal Facility at the National Institute of Health in Italy, in accordance with experimental protocols approved by the Italian Ministry of Health (ISS), following Legislative Decree 116/92 of the European Economic Community Directive 609/86.

#### 4.2.2. SOD1^G93A^ Transgenic Mice

SOD1^G93A^ transgenic mice were obtained by mating hemizygous B6SJL-Tg SOD1^G93A^ males (stock number 002726; RRID: IMSR_JAX:002726) purchased from The Jackson Laboratory (Bar Harbor, ME, USA) with C57BL/6J × SJL/J F1 hybrid females (strain name: B6SJLF1/JRj; RRID: IMSR_RJ:B6SJLF1) purchased from Janvier Labs (Saint-Berthevin Cedex, France). The offspring were identified by PCR assay as described in The Jackson Laboratory protocol. All of the mice were housed at the animal facilities of Universidad de Zaragoza under the following conditions: 12 h light/12 h dark cycle, 21–23 °C and a relative humidity of 55%. Food and water were available ad libitum. At the age of 60 and 120 days, 6 wild-type and 6 SOD1^G93A^ male mice were sacrificed by CO_2_ inhalation. Quadriceps muscles were harvested under sterile conditions. All procedures were carried out under Project License PI 08/19 approved by the in-house Ethics Committee for Animal Experiments from the University of Zaragoza on 27 March 2019, and following the international guidelines for the use of laboratory animals. The care and use of animals were performed according to the Spanish Policy for Animal Protection RD53/2013, which meets the European Union Directive 2010/63 on the protection of animals used for experimental and other scientific purposes.

### 4.3. RNA Extraction and Quantitative PCR

Muscle and cell culture samples were thawed at 4 °C and total RNA was isolated using the TRIzol reagent (15596026, Invitrogen, Carlsbad, CA, USA) following the manufacturer’s instructions. Briefly, the samples were incubated with chloroform and centrifuged to separate three phases, with the colorless upper aqueous phase being the only one collected. Subsequently, the RNA was precipitated using isopropanol and washed in 75% ethanol. Finally, the RNA was solubilized in RNase-free water and its concentration was quantified using the Nanodrop NS1000 spectrophotometer (Thermo Fisher Scientific, Waltham, MA, USA). Then, 500 ng of RNA was reverse transcribed using the High-Capacity cDNA Reverse Transcription Kit (Applied Biosystems, 4368814). The resulting cDNA was analyzed in triplicate by quantitative real-time polymerase chain reaction (qPCR) using the Quant Studio 3 Real-Time PCR Instrument (Applied Biosystems, Foster City, CA, USA). The customized self-designed Sybr Green Primers (Thermo Fisher Scientific, Waltham, MA, USA) used are detailed in Table A7. Target genes were normalized against glyceraldehyde 3-phosphate dehydrogenase (GAPDH) as the housekeeping gene and the relative gene expression was determined by the 2^−∆∆CT^ method [41], using threshold cycles (CT) from WT mice or C2C12 culture as control, depending on the assay.

### 4.4. Statistical Analysis

Results were statistically analyzed using GraphPad Prism 9 statistical software. Iterative Grubbs’ test was used to detect outliers that were excluded from the analysis. Parametric Student’s *t*-tests were used for comparisons between two groups in Assays 1, 3 and 4. Two-way univariate analysis of variance (ANOVA) and Bonferroni post hoc tests were performed in order to identify the effect of the genotype (C2C12/C2C12 MLC/SOD1^G93A^) and days of differentiation (Day0/Day2/Day5) in the expression of target genes and myogenin in the in vitro cultures (Assay 2). Data are shown as mean ± standard error of the mean (SEM), unless otherwise specified. For statistical significance, a threshold of 0.05 was selected and statistical significances were *p* ≤ 0.05 (*), *p* ≤ 0.01 (**), *p* ≤ 0.001 (***) and *p* ≤ 0.0001 (****). Given the targeted, hypothesis-driven nature of the eight-gene panel, we report nominal *p*-values. Results should be interpreted as exploratory and will require validation in larger cohorts.

## 5. Conclusions

In conclusion, our findings indicate that several sarcopenia-associated genes are transcriptionally active in skeletal muscle and display coordinated upregulation during physiological aging and at the early stages of ALS models. These results support the existence of a shared early molecular response characterized by inflammatory signaling, extracellular matrix remodeling, and tissue stress pathways. The convergence of these transcriptional changes suggests the emergence of a muscle aging-related molecular signature that may represent an early adaptive response to muscle degeneration. Further research will be needed to identify specific mechanisms affected as well as their potential as biomarkers and therapeutic targets to preserve muscle function and delay atrophy in both age-related and neurodegenerative contexts.

## Data Availability

All datasets generated and analyzed during the current study are available in Appendix A.

## Appendix A

**Table A1:** Average expression of genes’ threshold cycles in mice.

	*PF4*	*OIT3*	*MMRN1*	*MASP1*	*C1R*	*SVEP1*	*VCAN*	*SPTB*	*GAPDH*
**WT.5m.1**	25.3625	33.2465	31.2145	28.0595	26.7515	29.1945	27.9535	22.5605	18.7505
**WT.5m.2**	23.9867	31.6997	30.1737	26.2787	24.8977	27.1557	26.6387	22.4107	17.3407
**WT.5m.3**	24.8288	32.4731	29.2328	26.8198	25.6618	27.9398	27.5538	20.8678	17.2178
**WT.24m.1**	24.2860	32.2520	30.1700	28.7050	26.6780	28.6010	28.8530	22.6130	19.4940
**WT.24m.2**	24.5300	32.4840	29.3930	27.5310	26.5510	29.2470	28.6660	22.6440	19.9260
**WT.24m.3**	24.4080	32.7160	31.5180	28.7960	27.4270	28.5270	28.1890	22.7740	20.3730
**mSOD.5m.1**	24.0050	34.1390	29.7960	26.8720	25.5290	28.9310	27.0330	21.5600	18.0900
**mSOD.5m.2**	24.0800	33.6320	29.6020	26.1350	25.7280	28.8900	27.5930	21.7310	18.6160
**mSOD.5m.3**	24.5150	34.2270	29.6130	27.4330	25.4770	27.6300	26.8080	21.9500	18.9920
**mSOD.24m.1**	25.0840	No amp	30.3390	28.4630	27.1020	29.2540	28.0100	22.5990	20.0420
**mSOD.24m.2**	24.8330	No amp	30.5910	27.9190	27.4570	29.0190	29.0230	21.8070	19.6930
**mSOD.24m.3**	24.9770	No amp	30.2230	28.6250	26.7840	28.8340	28.7460	22.2900	19.7670
**WT.60d.1**	26.3760	33.4145	28.1501	25.5832	26.2862	26.2944	26.7412	22.3570	17.5224
**WT.60d.2**	27.2484	32.6963	28.0319	23.7829	26.0434	26.7605	26.6035	21.5713	16.9919
**WT.60d.3**	27.5829	33.5224	28.5424	24.5630	26.9720	27.5220	27.4077	21.7706	17.2179
**WT.60d.4**	26.5163	32.7960	29.4859	25.3294	26.8044	27.1540	26.8637	22.1783	16.9406
**WT.60d.5**	26.7201	33.4667	28.1430	23.8396	26.3448	25.7413	27.2194	21.6982	15.5936
**WT.60d.6**	26.7863	33.6724	29.1621	23.0798	26.3127	25.5872	26.7850	21.2954	15.9406
**SOD.60d.1**	27.3479	33.7706	29.1924	25.2230	26.7155	26.8833	27.7698	21.5636	16.9163
**SOD.60d.2**	27.7329	32.9742	28.8678	23.8825	26.7473	27.3594	27.7460	21.2126	17.3821
**SOD.60d.3**	27.5646	32.9349	28.5442	24.3575	26.3692	26.9563	27.3363	21.6244	17.5409
**SOD.60d.4**	27.1157	32.8686	28.3531	24.3846	26.4920	26.5763	27.3122	21.3498	18.0062
**SOD.60d.5**	27.2076	32.1309	28.0052	24.4160	26.1112	26.2145	26.7942	21.2485	18.3564
**SOD.60d.6**	26.7966	32.7555	27.9540	23.7824	25.9647	26.7122	26.4069	21.2856	17.0044
**WT.120d.1**	27.1145	33.3038	28.7469	23.9961	26.4615	26.9243	27.4887	21.7794	16.5173
**WT.120d.2**	26.7972	31.9822	27.9560	23.0795	25.9660	26.0741	27.2937	21.2337	16.5920
**WT.120d.3**	25.6073	34.9448	28.0416	24.2396	26.0930	26.2248	26.9574	21.1217	16.2788
**WT.120d.4**	25.3659	34.8670	28.3340	24.3859	26.1569	26.9195	26.9545	21.6499	16.7022
**WT.120d.5**	26.1854	34.7773	27.7491	24.1056	26.6576	27.1749	27.8385	21.3787	16.6054
**WT.120d.6**	26.1954	34.4617	27.4354	25.0067	26.4852	27.0313	27.7754	21.5907	17.0215
**SOD.120d.1**	28.6068	32.8386	29.5673	23.4594	26.5012	26.8329	27.1673	20.9606	17.3572
**SOD.120d.2**	27.4910	32.3179	29.5578	23.9753	26.3436	26.6403	28.6007	21.3765	18.0057
**SOD.120d.3**	26.9239	31.3480	28.0407	24.5536	26.4696	26.7889	27.8281	21.4856	16.4637
**SOD.120d.4**	27.1979	33.1795	28.9035	25.9727	26.1734	26.4937	28.0801	21.5991	16.3935
**SOD.120d.5**	26.0258	31.3321	27.9187	24.5430	25.8777	26.8362	26.2716	21.3296	16.5865
**SOD.120d.6**	26.4513	32.1662	28.2408	23.5651	25.8293	26.2508	26.7959	21.5371	16.2165

WT: Wild-type; mSOD: MLC/SOD1^G93A^ murine model; SOD: SOD1^G93A^ transgenic mice; No amp: no amplification.

**Table A2:** Results of the comparison between the expression levels of the target genes in the 5-month-old and 24-month-old WT mice from Assay 1.

Target Genes	ΔCT Mean of WT.5m	ΔCT Mean of WT.24m	*p* Value	Log2 Fold Change	2^−∆∆CT^ Mean of WT.5m	2^−∆∆CT^ Mean of WT.24m	SD Mean of WT.5m	SD Mean of WT.24m
***PF4***	6.956	4.698	0.013 *	2.258	1	4.784	0.675	0.912
***OIT3***	14.430	12.55	0.013 *	1.877	1	3.673	0.935	0.816
***MMRN1***	12.440	10.43	0.022 *	2.008	1	4.022	0.753	0.549
***MASP1***	9.283	8.413	0.158 ns	0.870	1	1.828	0.794	0.573
***C1R***	8.001	6.954	0.027 *	1.046	1	2.065	0.735	0.816
***SVEP1***	10.330	8.861	0.030 *	1.466	1	2.763	0.725	0.65
***VCAN***	9.612	8.638	0.167 ns	0.974	1	1.964	0.647	0.584
***SPTB***	4.177	2.746	0.044 *	1.431	1	2.696	0.583	0.779

Transcription levels of PF4, OIT3, MMRN1, MASP1, C1R, SVEP1, VCAN and SPTB were assessed in the gastrocnemius muscles of wild-type mice at the age of 5 months (WT.5m) and WT mice of 24 months old (WT.24m). Expression levels were evaluated by real-time PCR and parametric Student’s *t*-tests were used to compare the groups. *p* ≤ 0.05 (*).

**Table A3:** Results of the two-way ANOVA analysis and Bonferroni post hoc tests performed on target gene levels in different genotypes and differentiation times in Assay 2.

	Two-Way ANOVA of Matched Values			Bonferroni’s Multiple Comparisons Test				
***PF4***		**% of total variation**	***p*** **value**	**C2C12-CSOD**	**Mean Diff.**	**95.00% CI of diff.**	**Summary**	**Adjusted *p* value**
	**Differentiation**	32.27	1.69 × 10^−8^ ****	D0	−11.75	−14.50 to −9.003 ****		2.43 × 10^−5^
	**Genotype**	37.95	3.25 × 10^−9^ ****	D2	−7.292	−10.04 to −4.543 ***		3.77 × 10^−4^
	**Interaction**	29.7	2.16 × 10^−8^ ****	D5	−56.82	−59.57 to −54.07 ****		2.05 × 10^−9^
***OIT3***		**% of total variation**	***p*** **value**	**C2C12-CSOD**	**Mean Diff.**	**95.00% CI of diff.**	**Summary**	**Adjusted *p* value**
	**Differentiation**	1.127	0.957 ns	D0	0.735	−1.931 to 3.401 ns		>0.99
	**Genotype**	22.52	0.231 ns	D2	0.549	−2.117 to 3.215 ns		>0.99
	**Interaction**	0.3692	0.986 ns	D5	0.589	−2.077 to 3.255 ns		>0.99
***C1R***		**% of total variation**	***p*** **value**	**C2C12-CSOD**	**Mean Diff.**	**95.00% CI of diff.**	**Summary**	**Adjusted *p* value**
	**Differentiation**	45.88	3.66 × 10^−11^ ****	D0	−19.91	−22.79 to −17.03 ****		1.43 × 10^−6^
	**Genotype**	30.39	3.93 × 10^−11^ ****	D2	−21.66	−24.54 to −18.78 ****		8.66 × 10^−7^
	**Interaction**	23.71	2.65 × 10^−10^ ****	D5	−124.5	−127.4 to −121.6 ****		2.46 × 10^−11^
***VCAN***		**% of total variation**	***p*** **value**	**C2C12-CSOD**	**Mean Diff.**	**95.00% CI of diff.**	**Summary**	**Adjusted *p* value**
	**Differentiation**	19.21	0.009 **	D0	−1.877	−4.613 to 0.8585 ns		0.195
	**Genotype**	51.24	2.31 × 10^−4^ ***	D2	−1.952	−4.688 to 0.7835 ns		0.172
	**Interaction**	24.49	0.005 **	D5	−7.406	−10.14 to −4.670 ***		3.4 × 10^−4^
***SVEP1***		**% of total variation**	***p*** **value**	**C2C12-CSOD**	**Mean Diff.**	**95.00% CI of diff.**	**Summary**	**Adjusted *p* value**
	**Differentiation**	35.25	<1 × 10^−15^ ****	D0	−794.4	−797.3 to −791.6 ****		<1 × 10^−15^
	**Genotype**	32.38	<1 × 10^−15^ ****	D2	−1288	−1291 to −1285 ****		<1 × 10^−15^
	**Interaction**	32.37	<1 × 10^−15^ ****	D5	−8538	−8541 to −8535 ****		<1 × 10^−15^
***MASP1***		**% of total variation**	***p*** **value**	**C2C12-CSOD**	**Mean Diff.**	**95.00% CI of diff.**	**Summary**	**Adjusted *p* value**
	**Differentiation**	64.76	3.76 × 10^−6^ ****	D0	−2.165	−5.112 to 0.7816 ns		0.156
	**Genotype**	9.522	3.10 × 10^−4^ ***	D2	2.765	−0.1816 to 5.712 ns		0.06459
	**Interaction**	24.69	6.30 × 10^−5^ ****	D5	−12.2	−15.15 to −9.255 ****		2.93 × 10^−5^

Two-way ANOVA analysis and Bonferroni post hoc tests were performed to analyze the main effects of differentiation and genotype in transcriptional expression of PF4, OIT3, MASP1, C1R, SVEP1 and VCAN in mouse C2C12 MLC/SOD1^G93A^ (*CSOD*) and control (C2C12) cultures during myogenic differentiation. *p* ≤ 0.01 (**), *p* ≤ 0.001 (***) and *p* ≤ 0.0001 (****).

**Table A4:** Results of the statistical test performed on the expression levels of the target genes in the skeletal muscle of 5-month-old and 24-month-old WT and mSOD mice from Assay 3.

**Target Genes**	**ΔCT Mean of WT.5m**	**ΔCT Mean of mSOD.5m**	***p*** **Value**	**Log2 Fold Change**	**2^−∆∆CT^ Mean of WT.5m**	**2^−∆∆CT^ Mean of mSOD.5m**	**SD Mean of WT.5m**	**SD Mean of mSOD.5m**
***PF4***	6.956	5.634	0.008 **	1.322	1	2.501	0.675	0.844
***OIT3***	14.43	15.64	0.040 *	−1.215	1	0.498	0.935	0.686
***MMRN1***	12.44	11.1	0.007 **	1.333	1	2.519	0.753	0.682
***MASP1***	9.283	8.612	0.203 ns	0.672	1	1.593	0.794	0.846
***C1R***	8.001	7.012	0.040 *	0.989	1	1.984	0.735	0.715
***SVEP1***	10.33	9.918	0.382 ns	0.409	1	1.328	0.725	0.453
***VCAN***	9.612	8.579	0.033 *	1.034	1	2.047	0.647	0.633
***SPTB***	4.177	3.181	0.039 *	0.996	1	1.994	0.583	0.834
**Target Genes**	**ΔCT Mean of WT.24m**	**ΔCT Mean of mSOD.24m**	***p*** **Value**	**Log2 Fold Change**	**2^−∆∆CT^ Mean of WT.24m**	**2^−∆∆CT^ Mean of mSOD.24m**	**SD Mean of WT.24m**	**SD Mean of mSOD.24m**
***PF4***	5.236	5.131	0.855 ns	0.106	1	1.076	0.522	0.943
***OIT3***	12.55	-	- ns		1	-	0.816	-
***MMRN1***	10.43	10.55	0.831 ns	−0.121	1	0.92	0.549	0.806
***MASP1***	8.413	8.502	0.868 ns	−0.089	1	0.94	0.573	0.799
***C1R***	6.954	7.039	0.726 ns	−0.084	1	0.943	0.816	0.979
***SVEP1***	9.214	9.202	0.928 ns	0.012	1	1.009	0.9	0.914
***VCAN***	8.638	8.759	0.852 ns	−0.121	1	0.92	0.584	0.613
***SPTB***	2.746	2.398	0.239 ns	0.348	1	1.273	0.779	0.843

Parametric Student’s *t*-tests were used to compare transcription levels of PF4, OIT3, MMRN1, MASP1, C1R, SVEP1, VCAN and SPTB of each genotype and age. WT.5m: 5-month-old wild-type mice; mSOD.5m: 5-month-old MLC/SOD1^G93A^ mice; WT.24m: 24-month-old wild-type mice; mSOD.24m: 24-month-old MLC/SOD1^G93A^ mice. *p* ≤ 0.05 (*), *p* ≤ 0.01 (**).

**Table A5:** Statistical results of comparison in the expression levels of the target genes in the skeletal muscle of 60- and 120-day-old WT and SOD1^G93A^ mice from Assay 4.

**Target Genes**	**ΔCT Mean of WT.60d**	**ΔCT Mean of SOD1.60d**	***p*** **Value**	**Log2 Fold Change**	**2^−∆∆CT^ Mean of WT.60d**	**2^−∆∆CT^ Mean of SOD1.60d**	**SD Mean of WT.60d**	**SD Mean of SOD1.60d**
***PF4***	10.43	9.444	0.038 *	0.990	1	1.986	0.661	0.681
***OIT3***	16.56	15.07	0.022 *	1.485	1	2.8	0.506	0.574
***MMRN1***	12.14	10.69	0.025 *	1.449	1	2.731	0.529	0.613
***MASP1***	7.662	6.507	0.006 **	1.155	1	2.227	0.634	0.806
***C1R***	9.959	8.866	0.026 *	1.093	1	2.133	0.639	0.612
***SVEP1***	9.809	9.249	0.217 ns	0.559	1	1.474	0.673	0.552
***VCAN***	10.24	9.693	0.302 ns	0.542	1	1.456	0.545	0.555
***SPTB***	5.111	3.846	0.005 **	1.264	1	2.402	0.665	0.642
**Target Genes**	**ΔCT Mean of WT.120d**	**ΔCT Mean of SOD1.120d**	***p*** **Value**	**Log2 Fold Change**	**2^−∆∆CT^ Mean of WT.120d**	**2^−∆∆CT^ Mean of SOD1.120d**	**SD Mean of WT.120d**	**SD Mean of SOD1.120d**
***PF4***	9.591	10.28	0.126 ns	−0.688	1	0.621	0.613	0.607
***OIT3***	17.44	15.57	0.015 *	1.867	1	3.648	0.435	0.561
***MMRN1***	11.42	11.87	0.189 ns	−0.443	1	0.735	0.652	0.731
***MASP1***	7.516	7.093	0.432 ns	0.423	1	1.34	0.684	0.498
***C1R***	9.684	9.362	0.269 ns	0.322	1	1.25	0.818	0.663
***SVEP1***	10.11	9.803	0.354 ns	0.302	1	1.233	0.766	0.639
***VCAN***	10.77	10.62	0.695 ns	0.145	1	1.106	0.797	0.573
***SPTB***	4.84	4.544	0.444 ns	0.295	1	1.227	0.842	0.557

The transcription levels of PF4, OIT3, MMRN1, MASP1, C1R, SVEP1, VCAN and SPTB were compared by parametric Student’s *t*-tests. WT.60d: 60-day-old wild-type mice; SOD1.60d: 60-day-old SOD1^G93A^ mice; WT.120d: 120-day-old wild-type mice; SOD1.120d: 120-day-old SOD1^G93A^ mice. *p* ≤ 0.05 (*), *p* ≤ 0.01 (**).

**Table A6:** Supplementary data comprising all of the mice used in this study.

Name	Genotype	Sex	Age
**WT.5m.1**	WT	Male	5 months
**WT.5m.2**	WT	Male	5 months
**WT.5m.3**	WT	Male	5 months
**WT.24m.1**	WT	Male	24 months
**WT.24m.2**	WT	Male	24 months
**WT.24m.3**	WT	Male	24 months
**mSOD.5m.1**	MLC/SOD1^G93A^	Male	5 months
**mSOD.5m.2**	MLC/SOD1^G93A^	Male	5 months
**mSOD.5m.3**	MLC/SOD1^G93A^	Male	5 months
**mSOD.24m.1**	MLC/SOD1^G93A^	Male	24 months
**mSOD.24m.2**	MLC/SOD1^G93A^	Male	24 months
**mSOD.24m.3**	MLC/SOD1^G93A^	Male	24 months
**WT.60d.1**	WT	Male	60 days
**WT.60d.2**	WT	Male	60 days
**WT.60d.3**	WT	Male	60 days
**WT.60d.4**	WT	Male	60 days
**WT.60d.5**	WT	Male	60 days
**WT.60d.6**	WT	Male	60 days
**SOD.60d.1**	SOD1^G93A^	Male	60 days
**SOD.60d.2**	SOD1^G93A^	Male	60 days
**SOD.60d.3**	SOD1^G93A^	Male	60 days
**SOD.60d.4**	SOD1^G93A^	Male	60 days
**SOD.60d.5**	SOD1^G93A^	Male	60 days
**SOD.60d.6**	SOD1^G93A^	Male	60 days
**WT.120d.1**	WT	Male	120 days
**WT.120d.2**	WT	Male	120 days
**WT.120d.3**	WT	Male	120 days
**WT.120d.4**	WT	Male	120 days
**WT.120d.5**	WT	Male	120 days
**WT.120d.6**	WT	Male	120 days
**SOD.120d.1**	SOD1^G93A^	Male	120 days
**SOD.120d.2**	SOD1^G93A^	Male	120 days
**SOD.120d.3**	SOD1^G93A^	Male	120 days
**SOD.120d.4**	SOD1^G93A^	Male	120 days
**SOD.120d.5**	SOD1^G93A^	Male	120 days
**SOD.120d.6**	SOD1^G93A^	Male	120 days

WT: Wild-type; mSOD: MLC/SOD1^G93A^ murine model; SOD: SOD1^G93A^ transgenic mice.

**Table A7:** Primer sequences used for gene expression analysis.

Gene	Gene Name	Primer Sequence (5′-3′)	Annealing Temperature (°C)
**Housekeeping**			
***GAPDH***	Glyceraldehyde-3-phosphate dehydrogenase	F: AGGTCGGAGTCAACGGATTTG	60
		R: AGGGGTCATTGATGGCAACA	
**Target genes**			
***PF4***	Platelet factor 4	F: AGCTCATAGCCACCCTGAA	61.5
		R: AGGCAGCTGATACCTAACTCT	
***OIT3***	Oncoprotein induced transcript 3	F: GTGGTTGACGTGGTGAATGA	62
		R: CAGTTTGCTGGTCCGAATGA	
***MMRN1***	Multimerin 1	F: TGATGCAGTAGCACCAGATTT	62
		R: GTCATTCCGTGAGTGTGAGATAC	
***MASP1***	MBL associated serine protease 1	F: AGGAGAGGGAAGATGAAGAG	60
		R: TGTCTGTGTGGAGGATGT	
***C1R***	Complement C1r	F: GACCACCTATGAAGCCAGTATC	61.5
		R: GTATATATCCCTCGCATGGACTC	
***SVEP1***	Sushi, von Willebrand factor type A, EGF and pentraxin domain containing 1	F: ATGTTCCTGTAACCCACCATAC	61.5
		R: GCAGTACCCACGGTGTAAAT	
***VCAN***	Versican	F: CTGTGGATGGTGTTGTGTTTC	61.5
		R: TGCTCTGGGCTTGCTATG	
***SPTB***	Spectrin beta, erythrocytic	F: CACGCCGACCTTAACAAATG	61.5
		R: CCTCCACTCGCTTCAACTTA	
**Muscle differentiation marker**			
***MYOG***	Myogenin	F: AACTACCTTCCTGTCCACCTTCA	60
		R: GTCCCCAGTCCCTTTTCTTCCA	

F: Primer forward; R: primer reverse.
